# Targeting Inhibition of SmpB by Peptide Aptamer Attenuates the Virulence to Protect Zebrafish against *Aeromonas veronii* Infection

**DOI:** 10.3389/fmicb.2017.01766

**Published:** 2017-09-13

**Authors:** Peng Liu, Dongyi Huang, Xinwen Hu, Yanqiong Tang, Xiang Ma, Rihui Yan, Qian Han, Jianchun Guo, Yueling Zhang, Qun Sun, Zhu Liu

**Affiliations:** ^1^Hainan Key Laboratory for Sustainable Utilization of Tropical Bioresources, College of Biological Sciences, Hainan University Haikou, China; ^2^Institute of Tropical Bioscience and Biotechnology, Chinese Academy of Tropical Agricultural Sciences Haikou, China; ^3^Department of Biology, College of Science, Shantou University Shantou, China; ^4^Department of Biotechnology, College of Life Sciences, Sichuan University Chengdu, China

**Keywords:** *Aeromonas veronii*, SmpB, bacterial two-hybrid system, selection of peptide aptamers library, live attenuated vaccines

## Abstract

*Aeromonas veronii* is an important pathogen of aquatic animals, wherein Small protein B (SmpB) is required for pathogenesis by functioning as both a component in stalled-ribosome rescue and a transcription factor in upregulation of virulence gene *bvgS* expression. Here a specific peptide aptamer PA-1 was selected from peptide aptamer library by bacterial two-hybrid system employing pBT-SmpB as bait. The binding affinity between SmpB and PA-1 was evaluated using enzyme-linked immunosorbent assay. The key amino acids of SmpB that interact with PA-1 were identified. After PA-1 was introduced into *A. veronii*, the engineered strain designated as *A. veronii* (pN-PA-1) was more sensitive and grew slower under salt stress in comparison with wild type, as the disruption of SmpB by PA-1 resulted in significant transcription reductions of virulence-related genes. Consistent with these observations, *A. veronii* (pN-PA-1) was severely attenuated in model organism zebrafish, and vaccination of zebrafish with *A. veronii* (pN-PA-1) induced a strong antibody response. The vaccinated zebrafish were well protected against subsequent lethal challenges with virulent parental strain. Collectively, we propose that targeting inhibition of SmpB by peptide aptamer PA-1 possesses the desired qualities for a live attenuated vaccine against pathogenic *A. veronii*.

## Introduction

The aquaculture industry in China has been rapidly developed in recent years, and becomes one of leading parts in food supply (Lam et al., 2013). However, a large number of farmers adopt intensive culture in fish farms, giving rise to the deterioration of water quality and prevalence of diseases caused by viruses, bacteria and parasites (Mo et al., 2017). Particularly bacterial diseases outbreaks have resulted in tremendous economic loss in aquaculture industry (Peng et al., 2016).

*Aeromonas veronii* is a rod-shaped, motile, gram-negative bacterium that is distributed broadly in aquaculture environments (Li et al., 2011). As an opportunistic human-fish pathogen, *A. veronii* equips with several virulence factors, such as enterotoxin, haemolytic toxin, type three secretion effector AexU, the histidine kinases BvgS, serine protease, outer membrane protein and flagella (Li et al., 2011; Sreedharan et al., 2013). They cause the wound infection, diarrhea and septicemia in immune-compromised patients (Sun et al., 2016), and bacterial hemorrhagic septicemia in aquaculture animals (Li et al., 2011). For instance, *A. veronii* infects a broad range of fish, including yellow catfish (*Pelteobagrus fulvidraco*), channel Catfish (*Ictalurus punctatus*) and *Nile tilapia*, and subsequently results in the major economic losses (Kang et al., 2016; Dong et al., 2017; Yang et al., 2017).

In order to prevent and cure *A. veronii*, the antibiotics are widespread employed, thereby generating environmental contaminations, food safety problems, and the emergences of multidrug-resistant strains (Dhayanithi et al., 2015). Methods for bacterial disease prevention are extremely urgent and vaccines are considered as one of very promising tools. Previously, various formulations of vaccines with plasmid DNA, recombinant subunits and inactivated causative agents were applied for vaccines toward *A. veronii* (Reyes-Becerril et al., 2015). However, the referred agents show deficiency in productions, applications and poor immunogens, which leading to deficiencies of commercial vaccines for *A. veronii* species (Vazquez-Juarez et al., 2005). The live attenuated vaccines have been reported to be preliminary effective agents that mimic natural infection and stimulate a protective immune response, but they develop only as candidates for aquaculture at present and still have no commercial uses (Xiao et al., 2011; Zhang et al., 2012). Therefore, an effective and stable live attenuated vaccine is of great importance for application in aquaculture (Jiang et al., 2016).

During protein synthesis, the abnormal conditions generate loads of malformed mRNAs that lack appropriate termination signals, following with the stalled ribosomes on aberrant mRNAs (Dulebohn et al., 2007). This abnormality reduces the translational efficiency and produces aberrant proteins that might be deleterious for bacterial survival (Personne and Parish, 2014), therefore the rescue systems are needed for maintenances of cell viability.

Trans-translation mediated by transfer-messenger RNA (tmRNA) and Small protein B (SmpB) is the primary stalled-ribosome rescue system in bacteria in which SmpB functions as an essential component, to protect tmRNA from degradation, enhance tmRNA alanylation, and help tmRNA to bind with stalled ribosomes *in vivo* (Felden and Gillet, 2011). In addition, SmpB regulates both the RNA polymerase RpoS as a RNA chaperone (Liu et al., 2016) and the virulence sensor protein BvgS as a transcription factor (Liu et al., 2015), successively affecting protein synthesis, growth and adaptation to cellular stress, and pathogenic virulence. Recent reports show that *smpB* mutants serve as a live attenuated vaccine to provide effective immune protection. For instance, mice vaccinated with *smpB* mutants of *Francisella tularensis* or *Yersinia pestis* prevent infection from virulent wild type strains (Svetlanov et al., 2012).

Peptide aptamers are small combinatorial proteins that are selected to bind with specific molecules (Reverdatto et al., 2015). Peptide aptamers compose of 5–20 amino acids which fold as an exserted loop and embed into a stable protein scaffold. The conformation of surface loop is typically constrained, which results in high specificity and affinity with the target. Frequently the affinity with peptide aptamer disturbs the functions of the target protein and causes distinct phenotypes at intracellular level (Cobbert et al., 2015). Previously we constructed fabricated peptide aptamer libraries (pTRG-SN-peptides), which included both a scaffold protein *Staphylococcus aureus* nuclease (SN) and an loop consisted of random 16 amino acids (Liu et al., 2016). In this study, the conserved SmpB of *A. veronii* was considered as a potential antibacterial target. Because three ribosome rescue systems have been identified in bacteria, the alternative systems Arf A and Arf B are employed to rescue the ribosome by elevating their expression after the preferential *trans*-translation mediated by tmRNA and SmpB is deleted (Huter et al., 2017). To avoid the remedy of the ribosome rescue systems Arf A and Arf B, we tempted to use peptide aptamer to knock down the SmpB function, and successively reduced the virulence of *A. veronii* C4. This engineered strain possesses the property of a live attenuated vaccine, supporting a new strategy to prevent infection from *A. veronii* and fight against other pathogenic bacteria.

## Material And Methods

### Reagents and Chemicals

All Restriction endonucleases were purchased from New England BioLabs (NEB, Beijing, China). Pfu DNA Polymerase was purchased from Thermo Fisher Scientific (San Jose, CA, United States). All other reagents and chemicals were analytically pure grade from Takara (Otsu, Japan).

### Plasmid Constructions

All plasmids and primers used in this study were listed in **Table 1** and Supplementary Table S1, respectively. The truncations and mutants of pBT-SmpB and pN-SN were from our previous work (Liu et al., 2016). The peptide aptamer library (pTRG-SN-peptides) was constructed and comprised of approximate 2 × 10^7^ clones which expressed the scaffold protein and the random exposed loop (Liu et al., 2016). In brief, the DNA fragment encoding SN was inserted into pTRG, and expressed as a fusion protein with α-subunit of RNA polymerase as scaffold protein, in which the constrained loop composed of the residues S_63_L_64_R_65_K_66_A_67_ was replaced by 16 random amino acids. For construction of pET-28a-SmpB, the DNA fragment encoding SmpB was amplified from genomic DNA of *A. veronii* C4 using the primers F1/R1, at the end of which contained 5′-*Nco* I and 3′-*Xho* I restriction sites, and then digested and ligated into pET-28a to yield pET-28a-SmpB. For constructions of pET-28a-SN and pET-28a-PA-1, the DNA fragments were amplified using pTRG-SN or pTRG-PA-1 as templates and F2/R2 or F3/R3 as primers, respectively, followed by digestion and ligation with pET-28a. The plasmid pN-PA-1 was constructed using F4/R4 as primers according to our previous strategy (Liu et al., 2016).

**Table 1:** Plasmids used in this study.

Plasmids name	Description	Source or
		Reference
pBT-LGF2	Positive control, p15A ori, *lac*-UV-5 promoter, Cam^R^.	Stratagene
pTRG-Gal11^p^	Positive control, ColE1 ori, *lpp/lac*-UV5 promoter, Tet^R^.	Stratagene
pBT	Bait plasmid, p15A ori, *lac*-UV-5 promoter, Cam^R^.	Stratagene
pTRG	Prey plasmid, ColE1 ori, *lpp/lac*-UV5 promoter, Tet^R^.	Stratagene
pBT-SmpB	pBT derivative, expresses SmpB with λcI, Cam^R^.	Liu et al., 2015
pBT-SmpB ΔN34	pBT-SmpB derivative, deletes 34-residue at N-terminal of SmpB.	Liu et al., 2015
pBT-SmpB ΔN34C30	pBT-SmpB derivative, deletes 34-residue at N-terminal and 30-residue at C-terminal of SmpB.	Liu et al., 2015
pBT-SmpB ΔC30	pBT-SmpB derivative, deletes 30-residue at C-terminal of SmpB.	Liu et al., 2015
pBT (SmpB-G11S)	pBT-SmpB derivative, mutates G11S to AA.	Liu et al., 2015
pBT (SmpB-T14I)	pBT-SmpB derivative, mutates T14I to AA.	Liu et al., 2015
pBT (SmpB-F26I)	pBT-SmpB derivative, mutates F26I to AA.	Liu et al., 2015
pBT (SmpB-E32AG)	pBT-SmpB derivative, mutates E32AG to AAA.	Liu et al., 2015
pBT (SmpB-G133K)	pBT-SmpB derivative, mutates G133K to AA.	Liu et al., 2015
pBT (SmpB-D138KR)	pBT-SmpB derivative, mutates D138KR to AAA.	Liu et al., 2015
pBT (SmpB-K152)	pBT-SmpB derivative, mutates K152 to P.	Liu et al., 2015
pTRG-SN	pTRG derivative, expresses SN with RNAP, Tet^R^.	Liu et al., 2016
pTRG-SN-peptides	pTRG derivative, expresses random peptide with RNAP, Tet^R^.	Liu et al., 2016
pTRG-PA-1	pTRG derivative, expresses PA-1 with RNAP, Tet^R^.	This study
pET-28a-SmpB	pET-28a derivative, expresses SmpB, Kan^R^	This study
pET-28a-SN	pET-28a derivative, expresses SN, Kan^R^.	This study
pET-28a-PA-1	pET-28a derivative, expresses PA-1, Kan^R^.	This study
pN-SN	pRE112 derivative, expresses SN under the control of pk18mobsacB NEOKAN promoter.	Liu et al., 2016
pN-PA-1	pRE112 derivative, expresses PA-1 under the control of pk18mobsacB NEOKAN promoter.	This study


*Cam^*R*^, Tet^*R*^, and Kan^*R*^ denoted chloramphenicol, tetracycline, and kanamycin resistance, respectively.*

### Strains

Bacterial strains were listed in **Table 2**. *A. veronii*, *A. veronii* (pRE112), and *A. veronii* (pN-SN) were provided in our lab (Liu et al., 2016), and *A. veronii* (pN-PA-1) was constructed using the same method of *A. veronii* (pN-SN). *Escherichia coli* WM3064 was supplied as donor strain for genetic manipulation on pRE112 conjugative machinery in *A. veronii. E. coli* XL1-Blue MRF’ was applied to reproduce pBT and pTRG derivatives. *E. coli* XL1-Blue MR was used for bacterial two-hybrid system. *E. coli* BL21 (DE3) was used for the inducible expression of pET derivatives.

**Table 2:** Bacterial strains used in this study.

Strains name	Description	Source or
		Reference
*Aeromonas veronii* C4	Wild type, ampicillin resistance, virulent to *Ctenopharyngodon idella*.	Liu et al., 2015
*Aeromonas veronii* C4 (pRE112)	The engineered *A. veronii* C4 carries pRE112 empty vector.	Liu et al., 2016
*Aeromonas veronii* C4 (pN-SN)	The engineered *A. veronii* C4 expresses the SN by pN-SN recombinant plasmid.	Liu et al., 2016
*Aeromonas veronii* C4 (pN-PA-1)	The engineered *A. veronii* C4 expresses the PA-1 by pN-PA-1 recombinant plasmid.	This study
*E. coli* WM3064	*thrB1004 pro thi rpsL hsdS lacZ*ΔM15 RP4-1360 Δ(*araBAD)567*Δ*dapA1341::[erm pir]*.	Edwards et al., 1998
*E. coli* XL1-Blue MRF’	Δ*(mcrA)183*Δ*(mcrCB-hsdSMR-mrr)173 endA1 supE44 thi-1 recA1 gyrA96 relA1 lac* [F′*proAB lacIqZ*Δ*M15 Tn5* (Kan^r^)].	Stratagene
*E. coli* XL1-Blue MR	Δ*(mcrA)183*Δ*(mcrCB-hsdSMR-mrr)173 endA1 hisB supE44 thi-1 recA1 gyrA96 relA1 lac* [F′*lacIq HIS3 aadA* Kan^r^].	Stratagene
*E. coli* BL21(DE3)	*fhuA2 [lon] ompT gal* (λ *DE3) [dcm]*Δ*hsdS*λ *DE3* = λ *sBamHIo*Δ*EcoRI-B int::(lacI::PlacUV5::T7 gene1) i21*Δ*nin5*	NEB


### Selection of Peptide Aptamers (PA) and Identification of Interactive Sites between SmpB and PA

The plasmid pBT-SmpB was used as bait to screen specific peptide aptamers by Bacterial two-hybrid system. The peptide aptamer was selected and the interactive sites between SmpB and PA were identified as described previously (Liu et al., 2016).

### Expression and Purification of Recombinant Proteins

*Escherichia coli* strain BL21 (DE3) was transformed with pET-28a-SmpB, pET-28a-SN and pET-28a-PA-1, respectively. The strains were grown until OD_600_ of 0.4 in LB containing 50 μg/ml kanamycin, followed by supplementing with 0.1 mM isopropyl-β-d-thiogalactopyranoside (IPTG), and cultured overnight at 16°C. Cells were harvested, and resuspended in suspension buffer (10 mM PBS, pH 7.4) for sonication. The supernatant was collected and loaded onto nickel-iminodiacetic acid-agarose (Ni-IDA) column which was pre-balanced with equilibration buffer (Invitrogen, Frederick, MD, United States). Subsequently the column was washed with wash buffer (50 mM PBS, 10 mM imidazole, pH 7.4) until no further ultraviolet-absorbing values could be detected. Finally, the target protein was collected with elution buffer (50 mM PBS, 250 mM imidazole, pH 7.4), and estimated by SDS–PAGE. After the imidazole has been removed by dialysis, the concentration of protein was determined by BCA assay (Thermo Fisher Scientific, San Jose, CA, United States).

### Enzyme-Linked Immunosorbent Assay (ELISA)

The wells of enzyme-linked immunosorbent assay (ELISA) plate were coated with 100 μl of SmpB (100 μg/ml) at 4°C overnight. Concurrently, 3% BSA was chosen as the control. The following day the wells were washed three times with 200 μl of TTBS (20 mM Tris–HCl, pH 8.0, 0.05% Tween-20, 150 mM NaCl), and blocked at 37°C for 1 h with 200 μl of 3% BSA in PBS. The aliquots of SN or PA-1 (1.6 μM) were incubated with the wells which were pre-coated with SmpB at 4°C overnight. After the wells were washed three times in TTBS, the polyclonal rabbit antibody against 12 residues of SN was added into the wells at 37°C for 2 h, and subsequently anti-rabbit immunoglobulin G (IgG) was appended for 1 h, followed by the addition of 100 μl of TMB substrate reagent for 30 min and 100 μl of TMB termination buffer for cancellation. The absorbance at 450 nm was measured with Microplate Readers (BioTek, Winooski, VT, United States). Assays were performed in triplicate and the dissociation constants *K*_d_ were analyzed with GraphPad Prism version 6.0 (GraphPad, CA, United States).

### Homology Modeling and Protein–Protein Docking

The amino acid sequences of SmpB and PA-1 were aligned online using PROMALS3D, and optimal templates of SmpB (PDB code 1k8hA) and SN (PDB code 1jokA) were selected and applied to predict 3D-structures of SmpB and PA-1 by the iterative threading assembly refinement (I-TASSER) webserver (Yang et al., 2015). The most stable structures were projected for SmpB and PA-1 docking via the High Ambiguity Driven biomolecular DOCKing (HADDOCK) webserver^1^ (van Zundert et al., 2015). All the protein structures and docking complexes were visualized using the software PyMol Version 1.7.0.0.

### Growth Measurements in *A. veronii* C4 Derivatives

The plasmid pN-PA-1 was transformed into *E. coli* WM3064 and then transferred into *A. veronii* C4 by conjugation as described before (Liu et al., 2016). The growth curves of wild type *A. veronii* C4, *A. veronii* C4 (pRE112), *A. veronii* C4 (pN-SN) and *A. veronii* C4 (pN-PA-1) were measured with a UV-spectrophotometer (Mapada UV-1800, Shanghai, China) at regular intervals. The LB culture media were supplemented individually as follows: 2.5 mM CaCl2, 25 mM MgCl_2_ and 0.0–5.0% NaCl (Liu et al., 2016).

### Quantitative Real-Time PCR Analysis (qRT-PCR)

The wild type and engineered *A. veronii* C4 were grown to stationary phase in LB supplemented with either 50 μg/ml ampicillin or 50 μg/ml ampicillin and 25 μg/ml chloramphenicol simultaneously at 30°C. The total amount of RNA were extracted for relative expression analysis of genes, including *smpB*, three type secretion dependent effector (*aexU*), outer membrane protein (*ompA*), histidine kinases (*bvgS*), aerobactin (*aer*), serine protease (*ahp* gene), outer membrane channel (*tolC*), hemolysin (*trh*), low calcium response V (*lcrV*), RNA-binding protein (*hfq*), flagella basal body protein (*fliL*), universal stress protein A (*uspA*), and pilus assembly protein (*flpL*) (Liu et al., 2016). The primers of qRT-PCR were listed in Supplementary Table S2. The threshold cycle (*C*t) values of targets were normalized utilizing 16S rRNA as internal standard. And the relative expression quantity was calculated using the equation 2^-ΔΔ*C*_t_^, where ΔΔCt = (Ct target - Ct 16S rRNA)_Treatment_ - (Ct target - Ct 16S rRNA)_control_ (Liu et al., 2016).

### Determinations of 50% lethal Dose in Zebrafish

All animal experiments were approved by the Committee of the Ethics on Animal Care and Experiments at Hainan University, and all the animal experiments were carried out in accordance with the approved guidelines.

All zebrafish which were provided at the age of 4 months, average weight of ∼0.3 g and length of ∼3 cm, were fed with the basal diet and allowed to acclimate for at least 7 days before use. To evaluate 50% lethal dose (LD_50_), 10 zebrafish were intraperitoneally injected with 0.01 ml bacterial suspensions of wild type or *A. veronii* (pN-PA-1) in triplicate, and monitored at 25°C for 7 days, in comparison to the negative control with saline only. In the meantime, survival condition of zebrafish was recorded daily, and eventually LD_50_ was calculated by the method of Reed and Muench (1938). In brief, logLD50 = αlogβ+γ, where α = (The mortality higher than 50%-50%)/(The mortality higher than 50%-The mortality lower than 50%), β = dilution rate, in current experiment *b* = 10^-1^, γ = the log of minimum dilution rate, when the mortality higher than 50%.

### Measurement of IgM Antibody Levels in Zebrafish

The strains of *A. veronii* C4 (pN-PA-1) were grown in LB at 30°C, and harvested by centrifugation and re-suspension. The immunizations were consistently proceeded with three independent repeats, of which 20 zebrafish were injected with 1/10 LD_50_ *A. veronii* C4 (pN-PA-1) in the total amount of 1.62 × 10^6^ CFU/g, in contrast to negative control saline at 25°C. IgM antibody levels were determined in zebrafish sampled from 1 to 28 days by following the instructions of Fish IgM ELISA Kit (Mlbio, Shanghai, China). In brief, each sample including individual zebrafish was cut, weighed and frozen in liquid nitrogen and stored at -80°C for subsequent use. After the tissues were homogenized according to the proportion of 0.1 g per 1 mL PBS buffer (pH 7.4), the supernatant was collected. In the meanwhile, standards of purified IgM were diluted with TTBS buffer (containing 3% BSA) from 16 to 1 μg/mL using multiple proportion dilution method. Subsequently 50 μl of each standard or sample was loaded to the 96 micro-well plate pre-coated with an antibody specific for IgM for 30 min at 37°C. Each well was washed five times with TTBS, and incubated with 50 μl of diluted detection antibody for 30 min at 37°C. Having been washed five times, the plates were appended to 50 μl both of Chromogen Solution A and B for 15 min at 37°C. Eventually the absorbance at 450 nm was measured using microplate reader.

### Immunity and Protective Test

Having been vaccinated with 1/10 LD_50_ *A. veronii* (pN-PA-1) for 14 days, the challenge was conducted with 100 LD_50_ *A. veronii* C4 in the total amount of 4.98 × 10^7^ CFU/g. Mortality was examined, and dead zebrafish were removed in subsequent 7 days. The relative percent survival (RPS) was determined according to the following formula. RPS=[1-(% mortality of immunized fish/% mortality of control fish)]× 100 (Byon et al., 2005).

### Statistical Analysis

Statistical data were analyzed using the statistical Package for the Social Science (SPSS) version 20.0 (SPSS, Chicago, IL, United States) and GraphPad Prism version 6.0 (GraphPad, San Diego, CA, United States), and presented as mean values of three independent experiments with standard deviation (SD) using one-way analysis of variance (ANOVA). *P-*values less than 0.05 or 0.01 were considered as significant or extremely significant.

## Results

### Selection of Peptide Aptamers Interacting with SmpB by Bacterial Two-Hybrid System

**FIGURE 1:**
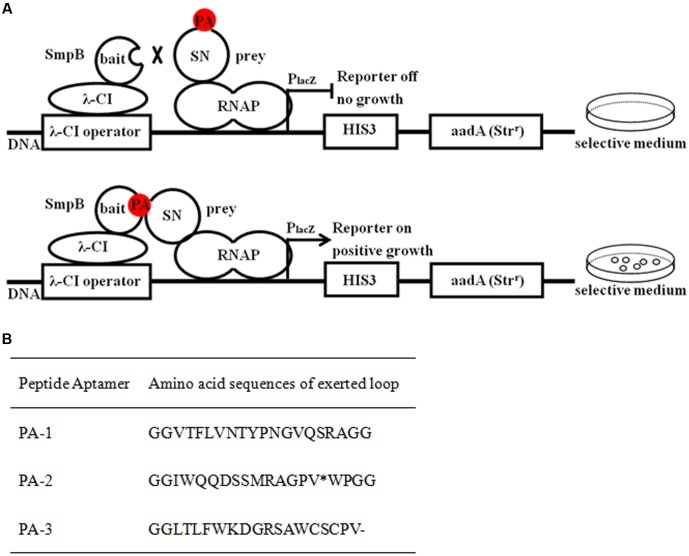
Selection of peptide aptamers interacting with SmpB by bacterial two-hybrid system. **(A)** Schematic illustration of selecting peptide aptamers which interact with SmpB. SmpB was fused to the full-length bacteriophage λ repressor protein (λ cI) as bait designating as pBT-SmpB, and correspondingly the peptide aptamers were fused to N-terminus of the α-subunit of RNA polymerase (RNAP) as preys designating as pTRG-SN-peptides. When SmpB and the specific peptide aptamers interacted, the transcription of the reporter gene would be activated and allowed bacterial growth on 5 mM 3-amino-1, 2, 4-triazole (3-AT) selective medium. **(B)** Sequence analyses of selected peptide aptamers. The single asterisk “^∗^” and dash “-” represented stop codon and frame shift mutations, respectively.

Bacterial two-hybrid selection was used to identify peptide aptamers that bound specifically to SmpB protein *in vivo* (**Figure 1A**). Three clones that might interact with SmpB were isolated from 2 × 10^2^ transformants and designated as PA-1, PA-2, and PA-3, respectively (**Figure 1B**). The sequencing results of the plasmids conferring the expressions of peptide aptamers revealed that PA-1 was the best candidate with correct open reading frame, while a stop codon existed in its encoded region of PA-2 and a frameshift mutation occurred in PA-3 (**Figure 1B**). The interaction between PA-1 and SmpB was confirmed again by Bacterial two-hybrid system (B2H) (Supplementary Figure S1).

### Evaluation of Binding Capacity between SmpB and PA-1

**FIGURE 2:**
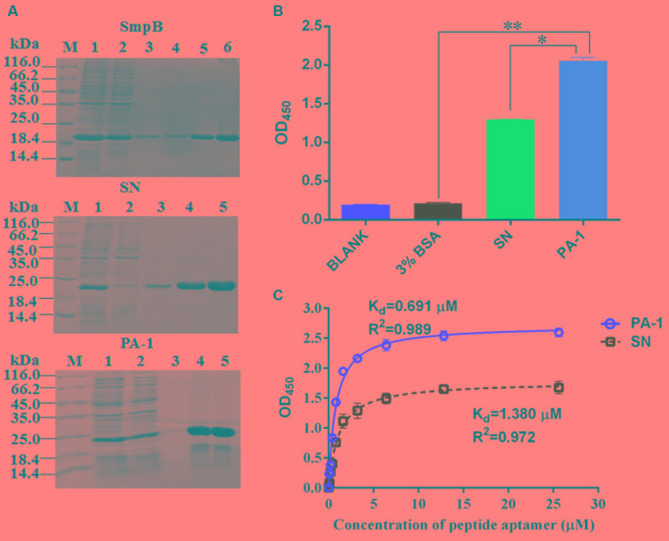
Evaluation of binding affinity between SmpB and PA-1. **(A)** SmpB, scaffold protein SN and PA-1 were cloned into the vector pET-28a and overexpressed in *E. coli* BL21 (DE3) cells. The purity of proteins was estimated by Coomassie Blue-stained SDS–PAGE. Lane M, molecular markers; Lane 1, the supernatant fraction of lysate; Lane 2, the flow-through fraction; Lane 3, the fraction washed by 10 mM imidazole (pH 7.4); Lane 4–6, the fractions eluted by 250 mM imidazole (pH 7.4). The expected molecular masses of His tagged-SmpB (Top), -SN (Middle) and -PA-1 (Bottom) were 19.4, 19.9, and 21.3 kDa, respectively. **(B)** Binding affinity analysis by ELISA. The final concentrations of PA-1, SN, BSA were set up at 1.6 μM, and SmpB was 2.6 μM. The results were represented as mean values of three independent experiments with standard deviation (SD). The single and double asterisk represented significant (*P* < 0.05) and extremely significant difference (*P* < 0.01). **(C)** Binding curves for SmpB and gradual increases of SN or PA-1 concentration.

The overexpressed SmpB, scaffold protein SN and PA-1 were purified on Ni-IDA column and verified using SDS–PAGE (**Figure 2A**). The ELISA was performed to evaluate binding affinity of SmpB with PA-1, SN or BSA (**Figure 2B**). PA-1 showed stronger interaction with SmpB than those controls of SN and BSA. The binding curve of PA-1 interacting with SmpB was plotted, and equilibrium dissociation constant (*K*_d_) was calculated by employing a model of one site binding-saturation analysis (Martinez-Archundia et al., 2012). The results showed that the binding of PA-1 to SmpB was stronger with *K*_d_ of 0.691 μM, in contrast to the binding of SN to SmpB with *K*_d_ of 1.380 μM (**Figure 2C**).

### Identification of Key Amino Acid Residues of SmpB Interacting with PA-1

**FIGURE 3:**
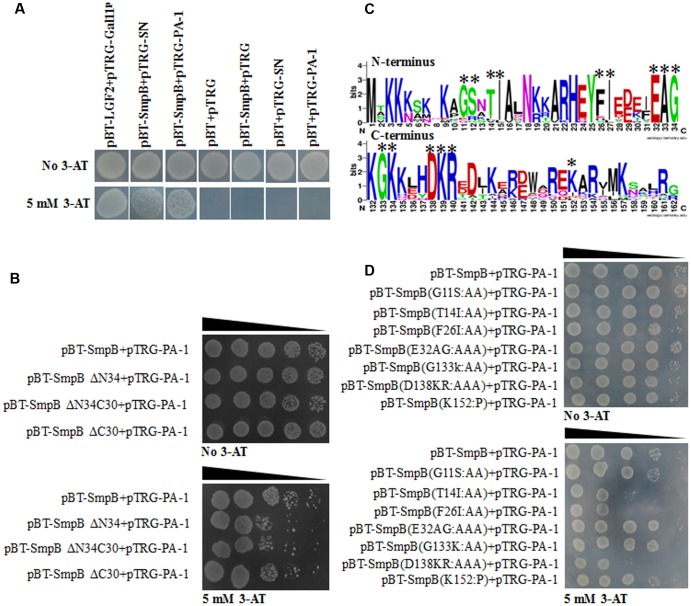
Identification of key amino acid residues of SmpB interacting with PA-1. **(A)** The co-transformants were cultivated overnight, and spotted onto no 3-AT and 5 mM 3-AT medium with 10 μl of 10-series dilution of initial 10 × 10^6^ CFU/ml. **(B)** Identification of key regions of SmpB interacting with PA-1 by Bacterial two-hybrid system. **(C)** Alignments of N- and C-terminal SmpB in different pathogenic bacteria by WebLogo 3 (http://weblogo.threeplusone.com/create.cgi). The mutated amino acids were marked with single asterisk. **(D)** Identification of key amino acid residues of SmpB interacting with PA-1 by Bacterial two-hybrid system.

Bacterial two-hybrid system was performed to study the interaction of SmpB and PA-1. Expectedly, SmpB and PA-1 had no self-activations and toxicities. Although scaffold protein SN interacted with SmpB, PA-1 displayed much stronger interplay with SmpB (**Figure 3A**). In order to further explore the region of SmpB to interact with PA-1, SmpB truncations including pBT-SmpB ΔN34, pBT-SmpB ΔN34C30 and pBT-SmpB ΔC30 were co-transformed with pTRG-PA-1, respectively. The results implied that N- and C-terminal residues of SmpB were required for its interaction with PA-1 (**Figure 3B**). Subsequently the conservative sites of N- and C-terminal SmpB were aligned from different pathogenic bacteria using WebLogo 3 (**Figure 3C**), and a series of pBT-SmpB mutants were constructed (Liu et al., 2015). When pTRG-PA-1 was co-transformed with pBT-SmpB (T_14_I_15_), pBT-SmpB (F_26_I_27_) and pBT-SmpB (D_138_K_139_R_140_), respectively, the cells showed growth defects on selective medium (**Figure 3D**), indicating the residues T_14_I_15_, F_26_I_27_ and D_138_K_139_R_140_ of SmpB were essential for the interaction with PA-1.

### Protein Modeling and Docking

**FIGURE 4:**
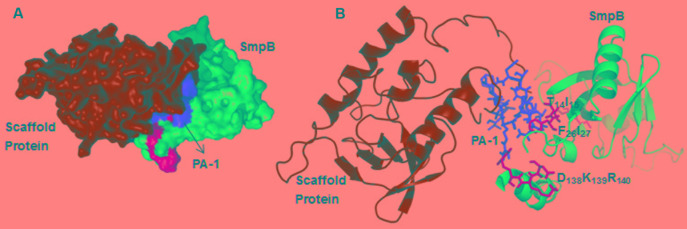
The docking results of SmpB and PA-1 complex. **(A)** SmpB, scaffold protein SN, the variable region of PA-1, and the residues T_14_I_15_, F_26_I_27_ and E_138_K_139_R_140_ were presented as a surface model with blue, green, red and yellow, respectively. **(B)** SmpB, SN, the variable region of PA-1, and the residues T_14_I_15_, F_26_I_27_ and E_138_K_139_R_140_ were presented as a ribbon model with blue, green, red and yellow, respectively.

Using HADDOCK docking program, the docking simulation was explored to verify the possible structural arrangements of PA-1 and SmpB complex, which were compatible with the previously identified residues from bacterial two-hybrid system. The HADDOCK between SmpB and PA-1 grouped the total of 146 structures into 9 clusters, which represented 80.3% of water-refined models, and the best model was selected when the lowest *Z*-score was -2.6. The docking result displayed that the residues T_14_I_15_, F_26_I_27_ and E_138_K_139_R_140_ interacted with the variable regions of PA-1 (**Figure 4**).

### PA-1 Inhibits SmpB Function *In Vivo*

**FIGURE 5:**
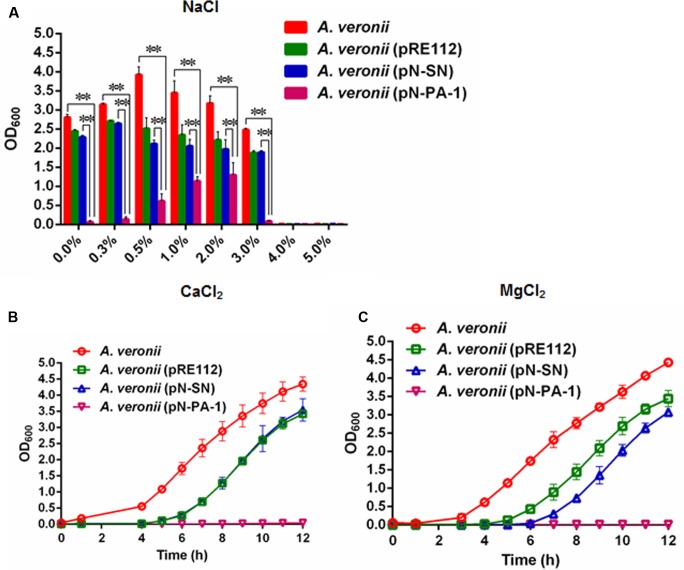
Growth of engineered *A. veronii* C4 strains at different salt stresses. **(A)** All the *A. veronii* C4 derivatives were grown overnight and diluted to an initial OD_600_ of 0.01 in LB medium appending different concentrations of NaCl (0.0–5.0%), and the samples were taken for OD_600_ measurement at 10 h. Besides, the cultures of engineered *A. veronii* C4 were supplemented with both 50 μg/ml ampicillin and 25 μg/ml chloramphenicol, except that wild type was only added 50 μg/ml ampicillin. **(B)** Growth curves of *A. veronii* C4 derivatives at 2.5 mM CaCl_2_. **(C)** Growth curves of *A. veronii* C4 derivatives at 25 mM MgCl_2_. The results were represented as mean values of three independent experiments with standard deviation (SD). The single and double asterisk represented significant (*P* < 0.05) and extremely significant difference (*P* < 0.01).

The plasmids pN-PA-1 and pN-SN were constructed and introduced into *A. veronii* C4 for evaluating the function of PA-1 according to our previous methods (Liu et al., 2016). When treated at different NaCl concentrations (0.0, 0.3, 0.5, 1.0, 2.0, 3.0, 4.0, and 5.0%), the engineered strain *A. veronii* C4 (pN-PA-1) expressing PA-1 showed severely impaired growth compared to wild type appearing best, and both of *A. veronii* C4 (pRE112) and *A. veronii* (pN-SN) manifesting intermediary growth. Under 4.0 and 5.0% NaCl concentrations, all the strains were not able to grow anymore (**Figure 5A**). At 2.5 mM CaCl_2_, the similar results were exhibited as demonstrated in 0–3.0% NaCl treatments (**Figure 5B**). At 25 mM MgCl_2_, the growth velocity was ranked as follows, wild type, *A. veronii* C4 (pRE112), *A. veronii* C4 (pN-SN) and *A. veronii* C4 (pN-PA-1), of which *A. veronii* C4 (pN-PA-1) did not grow (**Figure 5C**),while the growth differences were not evident in negative control LB medium (Supplementary Figure S2).

### Downregulations of Virulence Gene Transcriptions by Introducing PA-1 to *A. veronii* C4

**FIGURE 6:**
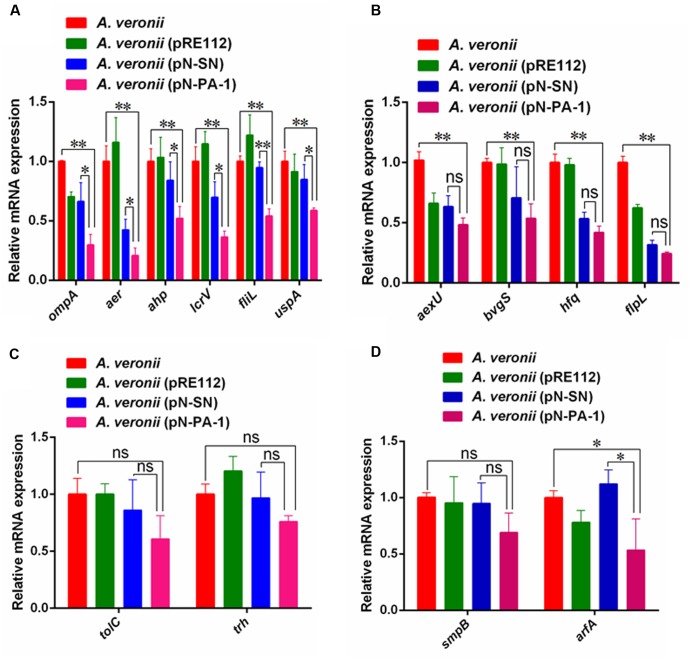
The qRT-PCR analysis of relative mRNA expression in *A. veronii* derivatives. **(A)** The relative expression analysis of *ompA*, *aer*, *ahp*, *lcrV*, *fliL* and *uspA*. In this group, the mRNA expression of these virulence genes in *A. veronii* C4 (pN-PA-1) had significant and extremely significant downregulation compared to *A. veronii* C4 (pN-SN) and wild-type, respectively. **(B)** The relative expression analysis of *aexU*, *bvgS*, *hfq* and *flpL*. In this group, the mRNA expressions of these virulence genes in *A. veronii* C4 (pN-PA-1) had extremely significant downregulation compared to wild-type, while they had no differences with *A. veronii* C4 (pN-SN). **(C)** The relative expression analysis of *tolC* and *trh*. In this group, the mRNA expressions of these virulence genes in *A. veronii* C4 (pN-PA-1) had no differences with wild-type and *A. veronii* C4 (pN-SN). **(D)** The relative expression analysis of *smpB* and *arfA*. In this group, the mRNA expressions of both ribosome rescue genes were compared. Error bars represented standard deviation from the mean values in triplicate. The single and double asterisk represented significant (*P* < 0.05) and extremely significant difference (*P* < 0.01).

After *A. veronii* C4 derivatives were grown to stationary phases, the total amount of RNA was extracted for relative expression analysis of virulence genes. The Quantitative Real-Time PCR (qRT-PCR) assays showed the transcriptional levels of *ompA*, *aer*, *ahp*, *lcrV*, *fliL* and *uspA* in *A. veronii* (pN-PA-1) were extremely significantly downregulated compared to wild-type *A. veronii* C4, and also showed significant differences with *A. veronii* (pN-SN) (**Figure 6A**). Besides, the levels of transcriptional downregulation of *aexU*, *bvgS*, *hfq* and *flpL* in *A. veronii* C4 (pN-PA-1) only had extremely significant differences compared to that of wild type (**Figure 6B**), while those of *tolC*, *trh* and *smpB* were identical among these strains (**Figures 6C,D**). The transcriptional level of *arfA* in *A. veronii* C4 (pN-PA-1) was downregulated significantly compared to those of wild type and *A. veronii* C4 (pN-SN) (**Figure 6D**), indicating not to compensate for the deficiency of SmpB function. Taken together, PA-1 interacted with SmpB and inhibited its function, thereby reducing the virulence gene expressions in *A. veronii* C4 (pN-PA-1).

### Immunization with *A. veronii* C4 (pN-PA-1) Protected Zebrafish against Subsequent Infection with Wild Type *A. veronii* C4

**FIGURE 7:**
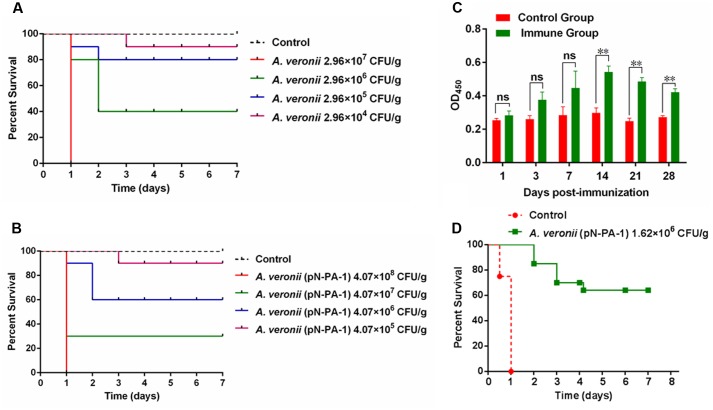
Immunization with *A. veronii* C4 (pN-PA-1) protected zebrafish against wild type *A. veronii* C4 challenge. **(A)** Determination of medium lethal dose (LD_50_ value) of wild type *A. veronii*. **(B)** Determination of medium lethal dose (LD_50_ value) of *A. veronii* C4 (pN-PA-1). **(C)** Measurement of IgM antibody levels in zebrafish vaccinated with *A. veronii* C4 (pN-PA-1) at 1, 3, 7, 14, 21, 28 days. **(D)** The relative percent survival (RPS) of zebrafish at 7 days. The results were represented as mean values of three independent experiments with SD. The single and double asterisk represented significant (*P* < 0.05) and extremely significant difference (*P* < 0.01).

To assess the medium lethal doses (LD_50_ value) of *A. veronii* C4 derivatives, the adult zebrafish were applied as the animal model. The survival numbers of zebrafish were recorded after wild type and *A. veronii* C4 (pN-PA-1) were injected with a series of appropriate doses. As a result, the LD_50_ value of wild type was 4.98 × 10^5^ CFU/g (**Figure 7A**), and the engineered strain *A. veronii* C4 (pN-PA-1) was 1.62 × 10^7^ CFU/g (**Figure 7B**), which was 33-fold higher than wild type. Next we investigated whether *A. veronii* C4 (pN-PA-1) could efficiently protect zebrafish against wild type attack. To explore whether *A. veronii* C4 (pN-PA-1) effectively stimulated fish immune response, the ELISA was performed to analyze the changes of immunoglobulin M (IgM) antibody level in zebrafish. The total tissues of zebrafish were collected for measuring IgM levels at different time points after vaccination. The result showed that the antibodies against IgM were significantly higher in the immune groups than the controls from 14 to 28 days (*P* < 0.01) (**Figure 7C**). Based on the varying patterns of IgM levels, a large amount of zebrafish were vaccinated with *A. veronii* C4 (pN-PA-1) for 14 days, and challenged with wild type *A. veronii* C4. The percent survival was recorded in the following 7 days, the vaccinated zebrafish were well protected with RPS of 65%, in contrast to 100% mortality of the control group which was injected with saline (**Figure 7D**).

## Discussion

Aquaculture industry in China currently encounters some problems, for example excessive aquaculture and overuse of antibiotics in farming procedures that have resulted in serious environment pollutions, antimicrobial drug residues, the emergences of multiple drug-resistant bacteria, and ultimately arousing a great threat to human health. Hence, the prevention and control of pathogenic bacteria must be developed in the aquaculture industry.

As the live attenuated vaccines are more efficient to prevent pathogenic bacteria by effectively stimulating protective immune responses than subunit vaccines or killed bacteria (Titball, 2008), they have been developed against bacterial fish pathogens including *Edwardsiellosis* (Xiao et al., 2011), *Streptococcus iniae* (Locke et al., 2008) and *Y. pestis* (Okan et al., 2010). And selection of specific virulence or vital function genes is the key for the construction of live attenuated vaccine. SmpB was chosen as the target for the construction of the live attenuated vaccine in *A. veronii* because of its prominent role in *trans*-translation (Sundermeier et al., 2005). In our work, the specific peptide aptamer PA-1 was screened to interact with SmpB (**Figure 1**). The PA-1 had strong binding affinity to SmpB with *K*_d_ of 0.691 μM, which was twofold higher than the control of SN binding to SmpB with *K*_d_ of 1.380 μM (**Figure 2**).

The key binding sites of SmpB T_14_I_15_, F_26_I_27_, E_138_K_139_R_140_ were also identified to interact with the exerted loop of PA-1 by bacterial two-hybrid system (**Figure 3**), in accordance with the display of molecular docking (**Figure 4**). The conserved sites E_138_K_139_R_140_ that are located on the C-terminal SmpB affected the rescue activity in the early stage of *trans*-translation (Kurita et al., 2010), and the hydrophobic residues T_14_I_15_, F_26_I_27_ located on the N-terminal SmpB were likely to have an effect on the structural formation (Dong et al., 2002).

The salinity tends to have an effect on the growth of *A. veronii* (Rael and Frankenberger, 1996), we wondered whether PA-1 abrogated SmpB functions by their interaction in *A. veronii* C4, resulting in more sensitivities. *A. veronii* C4 (pN-PA-1) showed extremely significant retardation of growth compared with other *A. veronii* C4 derivatives at 0.0–3.0% NaCl (**Figure 5A**). The growth of *A. veronii* C4 (pN-PA-1) was partly recovered in the range of 0.3–2% NaCl concentration, presumably due to the function compensation of alternative ribosome rescue factor ArfA. As shown in Supplementary Figure S3, the transcription level of *arfA* was elevated. When NaCl concentration was higher than 4.0%, the growth of all strains was completely suppressed, partially due to the membrane damages caused by the ultrahigh osmotic pressure (Pagán and Mackey, 2000).

Previous studies have shown that SmpB mutant had slower growth than wild type *Y. pestis* in LB supplemented with 2.5 mM CaCl_2_, as SmpB defects gave rise to the dysfunction of type three secretion system (T3SSs) that permitted to stress resistance in the presence of Ca^2+^ ions (Carlsson et al., 2007; Okan et al., 2010). Our results demonstrated that *A. veronii* C4 (pN-PA-1) had extremely slow growth rate in LB supplemented with 2.5 mM CaCl_2_ at 30°C, indicating that SmpB malfunction impaired T3SSs, and caused the growth defect (**Figure 5B**). This was confirmed by our RT-PCR results in which T3SSs-related genes *lcrV* and *aexU* were significantly downregulated (**Figures 6A,B**). The cation Mg^2+^ was reported to dissociate SmpB from tmRNA (Daher and Rueda, 2012), incurring tmRNA to lose the protection of SmpB, and thereinafter to be degraded by RNase R (Hong et al., 2005). Our data showed that *A. veronii* C4 (pN-PA-1) had an extremely slow growth and *A. veronii* C4 (pN-SN) only grew medially when they grew at 25 mM MgCl_2_ (**Figure 5C**). Taken together, the growth of *A. veronii* C4 (pN-PA-1) was seriously damaged at different salt stresses, indicating that PA-1 could recognize and inhibit SmpB functions in *A. veronii* C4.

In particular, since the relative mRNA transcription of *smpB* gene from different engineered strains was no significant difference, one reasonable interpretation was that PA-1 interacted with and disturbed SmpB at protein level instead of transcription level. However, the transcription of alternative ribosome-rescue factor A (*arfA*) had lower expression in *A. veronii* C4 (pN-PA-1) (**Figure 6D**), inconsistent with previous report that the enhancement of ArfA synthesis was regulated by *trans*-translation deletion (Schaub et al., 2012). We speculated that SmpB knockout could induce the upregulation of ArfA, whereas SmpB knockdown at protein level by PA-1 interaction would not stimulate ArfA to rescue stalled-ribosome. Since PA-1 could inhibit the expression of virulence factors indirectly and not stimulate the compensation of ribosome-rescue factor simultaneously, it seemed to be as a candidate for attenuated live vaccine.

The survival study revealed that the virulence of *A. veronii* C4 (pN-PA-1) was 33-fold attenuated compared to wild type (**Figures 7A,B**). The results were in accordance with the LD50 values of wild type and Hfq knockout, revealing 17-fold attenuation as a consequence of *hfq* gene deletion in *Vibrio alginolyticus* (Liu et al., 2011).

Furthermore, the specific antibody IgM from the tissue of vaccinated zebrafish was determined by ELISA in seven consecutive days, because IgM would respond strongly after vaccinated with pathogenic bacterium *V. anguillarum* in *Atlantic salmon* (Bøgwald et al., 1991). IgM is one of the most representative immunoglobulins (Ig), and commonly occurred during the immune response in fish (Bang et al., 1996). The results showed that IgM levels were increased gradually and maximized after 14 days of post-vaccination and the production of IgM extended significantly until 28 days compared to the control (**Figure 7C**), indicating that played an important role in the vaccine-induced protection. Also the transcription levels of immune-related genes IgM and IL-1β were determined by qRT-PCR, showing that immune-related genes had extremely significant differences compared with the negative control (Supplementary Figure S4).

The immunization with *A. veronii* C4 (pN-PA-1) provided 65% of protection rate in zebrafish (**Figure 7D**), better than DNA vaccinations of *Paralabrax maculatofasciatus* with outer-membrane protein genes from *A. veronii* (Vazquez-Juarez et al., 2005).

Although the virulence of *A. veronii* C4 (pN-PA-1) is attenuated and may be used as a potential live vaccine against *A. veronii* challenge, further questions need to be deciphered. Why does the pathogenicity become weakened in *smpB* knockdown? Does the reason come down to either its intracellular colonization defects or reduced escape from phagosomal compartment in macrophages? How does SmpB downregulate indirectly the virulence factors? Does it function as either transcriptional factor or stalled-ribosome rescued factor?

In summary, SmpB plays an important role in *A. veronii*, and the peptide aptamer PA-1 targeted to SmpB might knockdown its function. When PA-1 was transformed into *A. veronii*, the engineered strain could develop as a potential attenuated live vaccine, thereby providing a novel strategy to prevent *A. veronii* infection in aquaculture.

## Author Contributions

ZL and JG conceived and directed this study, designed the experiments, wrote and revised the manuscript. PL performed the experiments, analyzed the data and wrote the manuscript. DH and XH performed the experiments and analyzed the data. YT, XM, RY, QH analyzed the data and revised the manuscript. YZ and QS designed the experiments and revised the manuscript. All authors approved the manuscript to be published.

## Conflict of Interest Statement

The authors declare that the research was conducted in the absence of any commercial or financial relationships that could be construed as a potential conflict of interest.

## Footnotes

1 http://haddock.science.uu.nl/services/HADDOCK/haddockserver-easy.html
