# Supplementary material for: Targeting Inhibition of SmpB by Peptide Aptamer Attenuates the Virulence to Protect Zebrafish against Aeromonas veronii Infection
Source: Front Microbiol. 2017 Sep 13;8:1766. doi: 10.3389/fmicb.2017.01766 (PMC5601406; doi:10.3389/fmicb.2017.01766)
Supplement: Supplementary file 1 [file Table_1.DOCX]

Supplementary Material

**Targeting Inhibition of SmpB by Peptide Aptamer Attenuates the Virulence to Protect Zebrafish Against *Aeromonas Veronii* Infection**

**Peng Liu^1#^, Dongyi Huang^1#^,** **Xinwen Hu^1#^, Yanqiong Tang^1^,** **Xiang Ma ^1^, Rihui Yan^1^, Qian Han^1^, Jianchun Guo^2*^, Yueling Zhang^3^, Qun Sun^4^, Zhu Liu^1*^**

1 Hainan Key Laboratory for Sustainable Utilization of Tropical Bioresources/ College of Biological Sciences, Hainan University, Haikou 570228, China, 2 Institute of Tropical Bioscience and Biotechnology, Chinese Academy of Tropical Agricultural Sciences, Haikou 570711, China, 3 Department of Biology, College of Sciences, Shantou University, Shantou 515063, China, 4 Department of Biotechnology, College of Life Sciences, Sichuan University, Chengdu 610064, China

^#^ Peng Liu and Dongyi Huang and Xinwen Hu contributed equally to this work.

*** Correspondence:** Corresponding Author:

Zhu Liu, Ph.D., Professor

Jianchun Guo, Ph.D., Professor

Tel: 0086-898-66279014

E-mail: [zhuliu@hainu.edu.cn](mailto:zhuliu@hainu.edu.cn),
 jianchunguoh@163.com

# Supplementary Figures and Tables

## Supplementary Figure


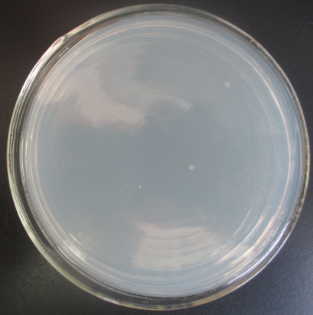


**2**

**3**


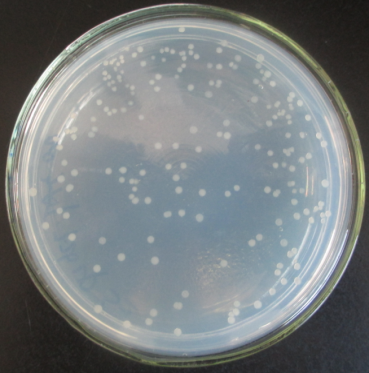


**No 3-AT**

**5 mM 3-AT**

**1**

**A**

**PA-1 no 3-AT**

**PA-1 5 mM 3-AT**


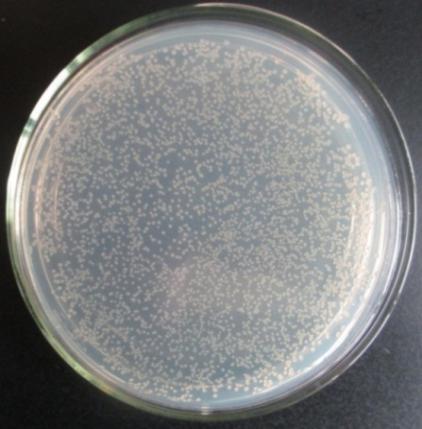

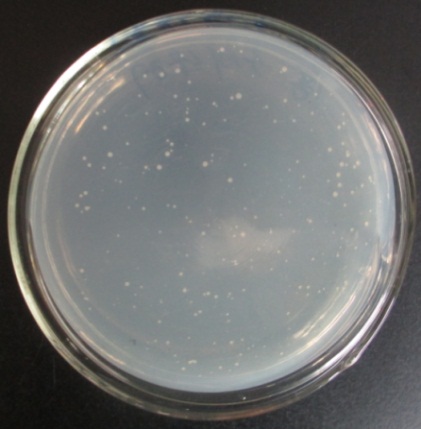


**B**

**Figure S1. Genetic Selection and identification of the specific peptide aptamer that interaction with SmpB by bacterial two-hybrid system. (A)** The interaction was monitored by growth on no 3-AT (left) and selective medium 5 mM 3-AT (right). **(B)** Confirmation of PA-1 interacts with SmpB by Bacterial two-hybrid system.

**
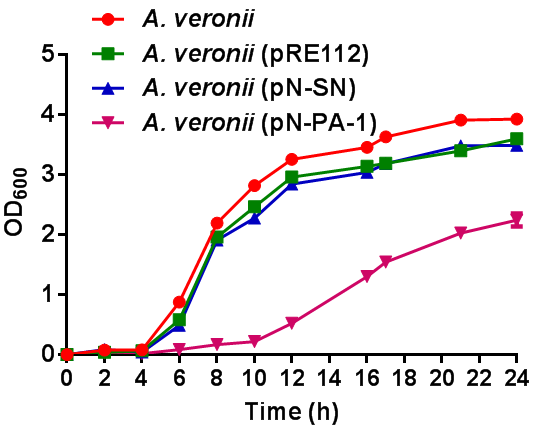
**

**Figure S2. Growth curves of *A. veronii* C4 derivatives in LB media.**


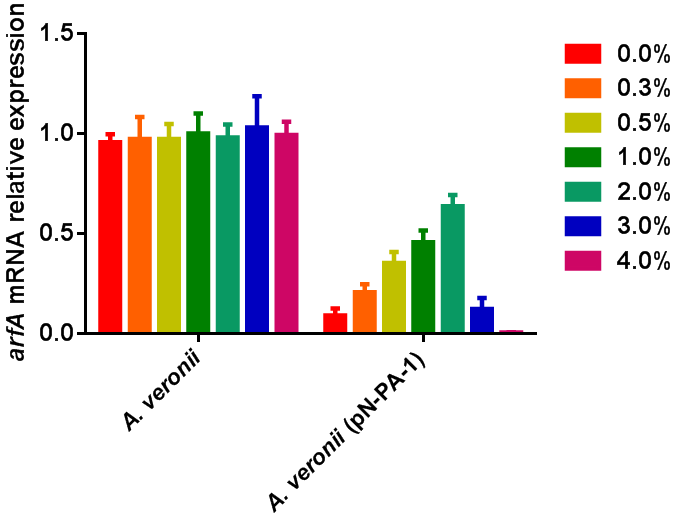


**Figure S3. The transcription level analysis of *arfA* under 0-4% NaCl concentrations in *A. veronii* C4 and *A. veronii* C4 (pN-PA-1).**


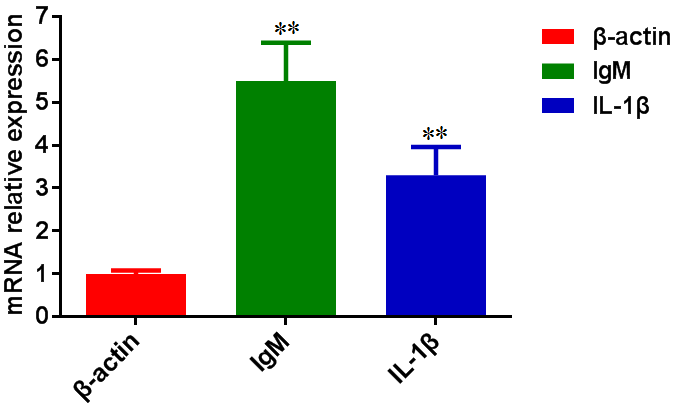


**Figure S4. Expression of immune-related genes in zebra ﬁsh vaccinated with *A. veronii* (pN-PA-1). The samples were taken at 14 days post-vaccination and used for qRT-PCR. The mRNA level of IgM and IL-1β was normalized to that of β-actin. For each gene, the mRNA level of the control ﬁsh was set as 1. Data were the means for triplicate experiments and presented as the mean ± SD. * P < 0.05, ** P < 0.01.**

## Supplementary Tables

**Table S1. Primers in this study.**

| **Primers** | **Sequnce(5’-3’)** | **Enzyme** |
| --- | --- | --- |
| pET-28a-SmpB  pET-28a-SN  pET-28a-PA-1    pN-PA-1 | F1:5’-CATGCCATGGGCAGCAAAAAAAACAGTAAAAACAAAGC-3’  R1:5’-CCGCTCGAGGCCGCGATGCTTGTTCTTCATG-3’  F2:5’-CATGCCATGGGCATGGGTTACCCATACGACGT-3’  R2:5’-CCGCTCGAGGTCGATGTCAACTTGACCAG-3’  F3:5’-CATGCCATGGGCATGGGTTACCCATACGACGT-3’  R3:5’-CCGCTCGAGGTCGATGTCAACTTGACCAG-3’  F4:5’-CCCCCGGGAGCGCAAAGAGAAAGCAGGTAG-3’  R4:5’-GCTCTAGAGGATCTCACTAGTTTAG-3’ | *Nco* I  *Xho* I  *Nco* I  *Xho* I  *Nco* I  *Xho* I  *Xma* I  *Xba* I |

**Table S2. Primers for real time PCR.**

| **Gene** | **Primers for Real-time PCR** |
| --- | --- |
| 16S rRNA | F5:5’-ATTCCAGGTGTAGCGGTGAA-3’  R5:5’-CACAGCCTCCAAATCGACAT-3’ |
| *smpB* | F6:5’-ATCGCTCGTCAGGGCTAC-3’  R6:5’-CGCTTGTCGTGCTCTTTC-3’ |
| *aexU*  *bvgS*  *ompA*  *aer*  *ahp*  *tolC*  *trh*  *lcrV*  *hfq*  *fliL*  *uspA*  *flpL*  *arfA*  β-actin  IgM  IL-1β | F7:5’-ATTGCCGATGGCTCCATCAA-3’  R7:5’-AGGGCATGACCAAGGGAAGA-3’  F8: 5’-GCACGTCTCTCTCCATCAAT-3’  R8: 5’-CATGTTGGCTAGTCGTAGTG-3’  F9: 5’-GATCGAAGGTGAAGCCAAGA-3’  R9: 5’-CAGGCATAGCCACCACCAAT-3’  F10:5’-TTATCAAGCCAGTCAGTTATCTC-3’  R10:5’-AGCCGCCGTCATTATTTC-3’  F11:5’-GAACACCTCCAACTACAA-3’  R11:5’-GTATAGAAGTCGGTCTTGATA-3’  F12:5’-CCTCTATCGTCGCAGTAAC-3’  R12:5’-GGTATCCATCGCCTTGAG-3’  F13:5’-CCTCTATCGTCGCAGTAAC-3’  R13:5’-GGTATCCATCGCCTTGAG-3’  F14:5’- ACGCAGGTCATCACAGTTA-3’  R14:5’-TGTTGTTGTCCTTGTCATAGC-3’  F15:5’-GATTGAGCAAGCCTTCTC-3’  R15:5’-CTGGTTACCATGAGCATC-3’  F16:5’-TTCCCTCTATGTCGGTATG-3’  R16:5’-CAAGGTGCCTTCAATCAG-3’  F17:5’-ATGAGTGGAACAACCTATTAC-3’  R17:5’-CGGTGTAGAGATCCTTGA-3’  F18:5’-GTCAACGCAAGGCATTCTG-3’  R18:5’-GGAGGCTTGGTGCTGTAA-3’  F18:5’-CAACTATGCCCATCAGCG-3’  R18:5’-CCTTGCCCTTCTTGTTGC-3’  F19:5’-ATGGATGAGGAAATCGCTGC-3’  R19:5’-CTCCCTGATGTCTGGGTCG-3’  F20:5’-GTGTTTGTGACTTGGCTTG-3’  R20:5’-CACCCGTCCACTCTGAATT-3’  F21:5’-TGGACTTCGCAGCACAAAATG-3’  R21:5’-GTTCACTTCACGCTCTTGGA-3’ |
